# Rhodopsin Mutant P23H Destabilizes Rod Photoreceptor Disk Membranes

**DOI:** 10.1371/journal.pone.0030101

**Published:** 2012-01-19

**Authors:** Mohammad Haeri, Barry E. Knox

**Affiliations:** Center for Vision Research, Departments of Neuroscience and Physiology, Biochemistry and Molecular Biology and Ophthalmology, State University of New York Upstate Medical University, Syracuse, New York, United States of America; University of Oldenburg, Germany

## Abstract

Mutations in rhodopsin cause retinitis pigmentosa in humans and retinal degeneration in a multitude of other animals. We utilized high-resolution live imaging of the large rod photoreceptors from transgenic frogs (*Xenopus*) to compare the properties of fluorescently tagged rhodopsin, Rho-EGFP, and Rho^P23H^-EGFP. The mutant was abnormally distributed both in the inner and outer segments (OS), accumulating in the OS to a concentration of ∼0.1% compared to endogenous opsin. Rho^P23H^-EGFP formed dense fluorescent foci, with concentrations of mutant protein up to ten times higher than other regions. Wild-type transgenic Rho-EGFP did not concentrate in OS foci when co-expressed in the same rod with Rho^P23H^-EGFP. Outer segment regions containing fluorescent foci were refractory to fluorescence recovery after photobleaching, while foci in the inner segment exhibited recovery kinetics similar to OS regions without foci and Rho-EGFP. The Rho^P23H^-EGFP foci were often in older, more distal OS disks. Electron micrographs of OS revealed abnormal disk membranes, with the regular disk bilayers broken into vesiculotubular structures. Furthermore, we observed similar OS disturbances in transgenic mice expressing Rho^P23H^, suggesting such structures are a general consequence of mutant expression. Together these results show that mutant opsin disrupts OS disks, destabilizing the outer segment possibly via the formation of aggregates. This may render rods susceptible to mechanical injury or compromise OS function, contributing to photoreceptor loss.

## Introduction

The most common allele in North America for autosomal dominant retinitis pigmentosa, a major cause of retinal degeneration and vision loss worldwide [1], is a missense mutation (P23H) in the gene encoding rhodopsin [2]. The molecular pathophysiology that leads to eventual photoreceptor death is unclear. The accumulation of misfolded Rho^P23H^ in the endoplasmic reticulum was proposed to cause cellular stress [3], however strong support in photoreceptors is lacking. A common feature of mechanisms proposed to explain cell loss in retinitis pigmentosa (RP) is misfolded protein retention and eventual aggregation in the endoplasmic reticulum (ER) inducing severe cellular stress [4]. In several different cultured mammalian cell lines [3], [5], Rho^P23H^ was prominent in the cytoplasm and formed aggregates resembling inclusion bodies and aggresomes similar to those described in other diseases such as cystic fibrosis (CFTR) and the early-onset familial Alzheimer's disease (PS1) [6]. Rho^P23H^ causes loss of rods and retinal degeneration in many different animal models including mouse [7]–[9], rat [10], [11], pig [12], frog [13] and even in flies [14]. A serious complication is the gene dosage effect, since relative protein levels of Rho^P23H^ and wild type rhodopsin influence rod degeneration [7], [13], [15]. Studies of the pathogenic mechanism have focused on the cellular distribution of Rho^P23H^. In some animal models, rhodopsin has been found in both inner and outer segments [13], [15], although this has not been observed in Rho^P23H^ knock-in mice [9]. All animal models show a disturbance of the OS in terms of length, with eventual OS disorganization as rods die. However, the cause of the cellular toxicity has not been identified. We have investigated whether protein aggregates, the central premise of pathological mechanism in neurodegenerative diseases [16], occur in rods expressing Rho^P23H^. We have quantified the distribution and properties of a Rho^P23H^-EGFP fusion protein in transgenic *Xenopus*, a system well-suited for live cell imaging. Focusing on rods with low fluorescent protein expression levels has allowed us to characterize primary properties of the mutant protein and avoid the complications of overexpression.

## Results and Discussion

**Figure 1:**
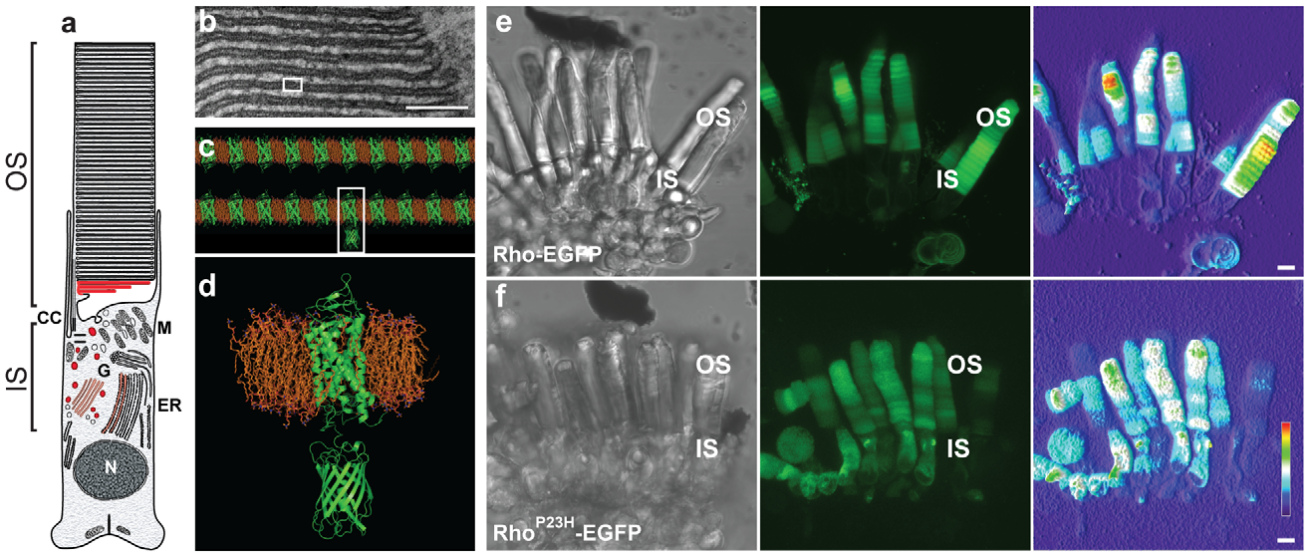
Expression of Rho^P23H^-EGFP in transgenic *Xenopus*. (**a**) Schematic drawing (*left*) of the *Xenopus* rod photoreceptor. The inner (IS) and outer (OS) segments, the nucleus (N), the prominent endoplasmic reticulum (ER), Golgi (G), connecting cilium (CC) and mitochondria (M) located apically in the IS are shown. The OS contains numerous stacks of disk membranes. (**b**) A segment of OS is shown in an electron micrograph. Scale bar, 100 nm. (**c**) A molecular model of the OS disk membrane (from a segment of a single disk, *white box in b*). The density of rhodopsin, approximately 90% of the protein in the OS disk membranes [32] is illustrated to scale in the molecular homology model based upon the high-resolution bovine rhodopsin structure [33]. The rhodopsin-phospholipid molar ratio is presented to scale. (**e, f**) Representative images of sections of live *Xenopus* retina showing rod cells expressing either Rho-EGFP (e) or Rho^P23H^-EGFP (f) The DIC image of a small piece of retina (*left*) and the corresponding three dimensional rendering of a confocal laser scanning z-stack using EGFP detection (*middle*) are shown. The outer and inner segments are labeled. To illustrate the range of transgene expression, a concentration heat map (red, maximum intensity) is shown (*right*). Scale bar, 5 µm.

**Figure 2:**
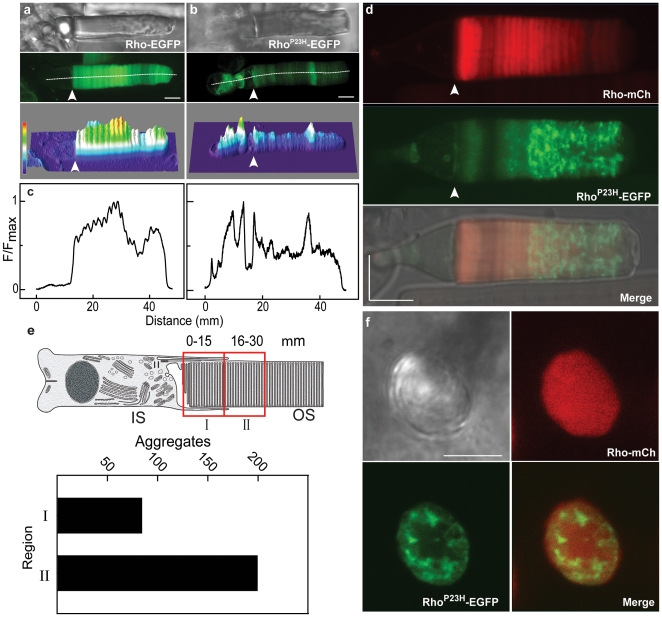
Aberrant expression and aggregation of Rho^P23H^-EGFP in rod photoreceptors. Confocal images of representative live cells expressing rhodopsin-EGFP fusion proteins show distributions of Rho-EGFP (**a**) and mutant opsin, Rho^P23H^-EGFP (**b**). DIC (*top*) and fluorescence (*middle*) images were used to calculate the fluorescence distribution, displayed as a heat map (*bottom*): red for most intense, green for mid-level and blue for least intense. The border between the IS and OS is indicated by the arrow. (**c**) The fluorescence profile distribution was computed along the z-spline path through the center of the cell (*white line*), with the origin arbitrarily set in the nuclear region for each cell. The fluorescence intensity was normalized to the maximum value along the spline. (**d**) High resolution images of a representative live rod expressing fluorescent protein from two rhodopsin cassettes: Rho-mCherry (*red*, encoding wild type opsin) and Rho^P23H^-EGFP (*green*). The fluorescence intensity profile of two transgenes was determined simultaneously, and the individual distributions (mCherry, *top* and EGFP, *middle* are shown together with the distributions merged onto a DIC image (*bottom*). (**e**) Quantification of the fluorescent foci in two OS axial locations in live rods expressing Rho^P23H^-EGFP. The OS was divided into two sections (I and II), comprising ∼60% of the length. The more distal region of the OS was not included to avoid regions prone to swelling or other *in vitro* damage. The number of isolated fluorescent foci in the outer segment regions was counted in a total of 27 cells from 16 transgenic tadpoles expressing Rho^P23H^-EGFP. (**f**) Representative cross-sectional view of an OS from a live rod expressing two rhodopsin cassettes: Rho-mCherry and Rho^P23H^-EGFP. The *z*-section for the CSLM was parallel to the rod axis, and the fluorescence from each channel is shown separately and merged. Scale bars, 5 µm.

**Figure 3:**
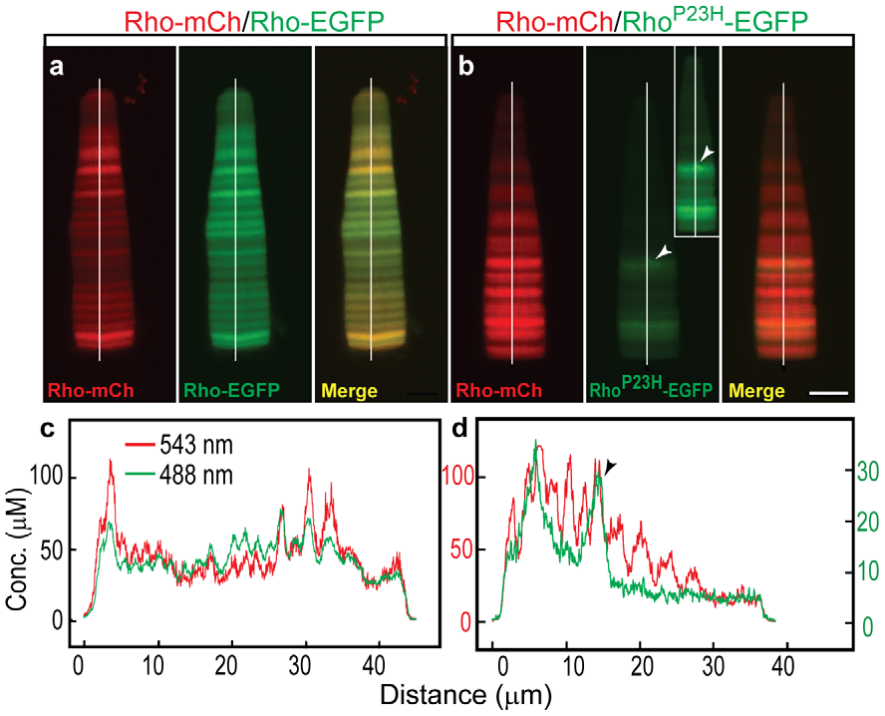
Quantification of transgene expression from dual rhodopsin cassettes in *Xenopus* rods. (**a, b**) Measurement of the EGFP and mCherry fluorescence distributions in cells expressing Rho-mCherry/Rho-EGFP (a) and Rho-mCherry-Rho^P23H^-EGFP (b). Control rods with no fluorescent protein had only background fluorescence which was set to zero. Images show the mCherry (*red*), EGFP (*green*) and merged distributions for representative rods. The merged image (a, *right*) shows synchronized changes in the expression level of both transgenes (Rho- EGFP and Rho-mCherry). The concentration of both proteins is comparable along the rod axis. (b) The Rho^P23H^-EGFP expression level is significantly lower (b, *middle*) than the co-expressed wild type Rho-mCherry (b, *left*). The inset in panel (b, *middle*) shows the intensified EGFP channel of the original figure to clarify its profile. (**c, d**) Distributions were measured along the central axis of the cell (*white line*), converted to concentration using the calibration in Fig. S3 and plotted as a function of distance from the OS base. The dual wild type cassettes are shown in (c) at the same scale for both green and red channels, while for the dual cassette containing Rho^P23H^EGFP (d), the EGFP values (*green*) are given on right Y-axis (*green*) and the mCherry values (*red*) on left Y-axis. The distance is measured from the base of the rod OS. The arrowhead indicates fluorescent foci in the Rho^P23H^-EGFP rod. Scale bar, 5 µm.

Quantitative analysis of Rho-EGFP and Rho^P23H^-EGFP distributions in transgenic *Xenopus* rods showed a marked difference between the two fusion proteins (Fig. 1,2,3 and Fig. S1). Rho-EGFP localized primarily in the OS of mature rods, while Rho^P23H^-EGFP was distributed in both OS and inner segments (IS), localizing around the nucleus and more diffusely in the ellipsoid and myoid areas but not in the synaptic region. The retinal phenotype from the Rho^P23H^-EGFP depended strongly on the expression level of the transgene, with the most severe effects in those retina with highest concentration of Rho^P23H^-EGFP in rods prior to degeneration (Fig. S1, see also [13]). These include shortening and loss of the OS, mislocalization in the IS and abnormal shedding events. In order to investigate the intrinsic properties of Rho^P23H^-EGFP in non-degenerating rods, we focused on those transgenic animals with expression levels that were less than ∼5% of the endogenous level in both primary transgenic animals and different transgenic lines (Table 1). We note that there was a significant attenuation of Rho^P23H^-EGFP expression levels over several weeks in all animals. This reduction in expression level was not accompanied by a loss in rod number. Comparable reduction in transgene expression was not observed in animals expressing either Rho-EGFP (Fig. S1) or even soluble EGFP (*data not shown*) even though expression levels were much higher.

**Table 1:** Quantification of rhodopsin-EGFP transgenes in Xenopus rods.

	Outer Segment (mM)	Ratio OS/IS
**1. Rhodopsin (endogenous)**	3*	>100§
**2. Rho-EGFP (F_0_)**		
Mean (n = 39)	0.11	613
SD	0.049	
**3. Rho^P23H^-EGFP (F_0_)**		
Mean (n = 25)	0.0091	44.4
SD	0.0052	
**Dual Transgenic (F_1_)**		
**4. Rho-mCherry**		
Mean (n = 23)	0.028	N.D.
SD	0.014	
**5. Rho^P23H^-EGFP**		
Mean (n = 23)	0.004	N.D.
SD	0.003	

*[17].

§ Estimated from serial section western blot data [34].

To directly compare the behavior of Rho and Rho^P23H^ in the same cell, transgenic animals were produced using dual transgene expression constructs consisting of two rhodopsin cassettes in tandem. Both Rho-EGFP and Rho-mCherry exhibited a regular transverse banding in the OS (Fig. 2a, d) that is related to the diurnal variation in rhodopsin density in the OS membranes (Haeri *et al.*, *in preparation*). However, mutant opsin exhibited abnormal foci that contained high concentrations of fluorescent mutant protein in both the IS and OS (Fig. 2b). Foci were observed independent of the fluorescent protein fused to its carboxyl terminus (EGFP, Fig. 2 and mCherry, Fig. S5). The wild type protein did not show a co-concentration with the mutant fluorescent foci (Fig. 2d). Rods expressing Rho^P23H^-EGFP imaged along the axial length of the OS showed fluorescent foci in the mid-periphery of OS disks (Fig. 2f). This is in contrast to the behavior of Rho-EGFP that was found throughout the axial optical section (Fig. 2f), but was typically at much reduced concentration in disk incisures (Fig. S4). This reduction of Rho^P23H^-EGFP from disk incisures was not observed (Fig. 2). Quantitative analysis of fluorescent protein concentrations strongly suggests that foci formation is specific to the mutant protein, since the concentration of Rho^P23H^-EGFP is much lower than the co-expressed wild type transgene (Figs. 2 and 3) and endogenous rhodopsin (Table 1). It is important to note that although the transcript levels for Rho^P23H^-EGFP and co-expressed Rho-mCherry are very similar (Fig. S2), the protein concentrations are very different (Table 1 and Fig. 3). Thus, there is either increased protein degradation or decreased translation of the Rho^P23H^-EGFP transcript, or both, in mutant-expressing rods. A similar finding has been reported for knock-in Rho^P23H^ mice [9].

Daily membrane biosynthesis adds approximately 80 disks at the base of *Xenopus* OS every day, which is equivalent to ∼1.2 µm in OS length [17]. Thus, the OS contains a record of the rhodopsin biosynthesis over more than 4 weeks, with the oldest disks at the apical end. We quantified the number of foci in two regions of the OS, base and central. We restricted the analysis to the proximal half of the OS to minimize *in vitro* artifacts introduced by OS damage, *e.g.* breakage or swelling. There were a highly variable number of foci found per OS, from absent to more than 20. It is important to note that the experiments reported here did not characterize fluorescent foci less than 0.4 µm. The foci had different shapes and sizes, but it was possible to count the number of distinct foci per OS. We found more fluorescent foci in the central region than in the basal region, *i.e.* in older OS membranes (Fig. 2e). These results suggest that the probability of forming foci or having them reach a detectable size may be a slow process.

**Figure 4:**
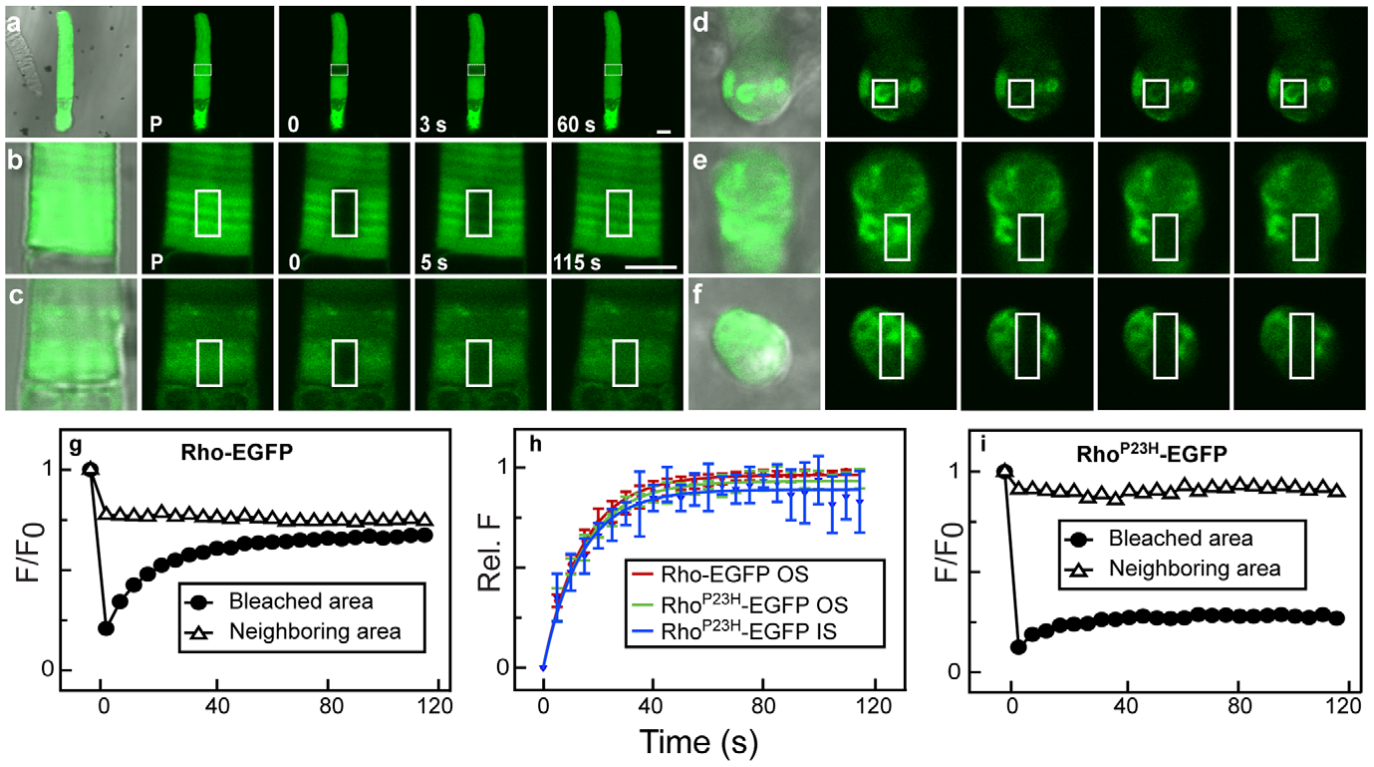
Aberrant mobility of Rho^P23H^-EGFP measured using fluorescence recovery after photobleaching. (**a**) Representative low magnification CSLM images of a live rod expressing soluble EGFP show the fluorescence distribution (merged with the DIC image, *left*) and with the region of interest (ROI) indicated (*white box*). The cell (*P*, pretreatment) was photobleached in the ROI (0 s) and the recovery was followed for the indicated time (*in seconds*). (**b–f**) Live rods expressing Rho-EGFP or Rho^P23H^-EGFP were photobleached in different regions and images from different times in the recovery period are shown at higher magnification. The pre-bleach sample is shown as a merge (fluorescence with DIC, *left*) and with fluorescence alone (*P*) followed by representative images immediately after bleach (0 s) and 5 s and 115 s post-bleach. The ROI is boxed in each image. Various compartments of the cells were photobleached: (**b**) base of the OS in cells expressing Rho-EGFP; (**c**) near the base of the OS in a region with no fluorescent foci in a cell expressing Rho^P23H^-EGFP; (**d**) IS in a cell expressing Rho^P23H^-EGFP; (**e**) in the OS with fluorescent Rho^P23H^-EGFP foci; (**f**) in an axial view of the OS with fluorescent Rho^P23H^-EGFP foci. (**g**) The photobleaching recovery profile of panel (b). Integrated fluorescence of Rho-EGFP in the ROI defined in (b), normalized to the integrated fluorescence of the same region in the pre-photobleached image (*closed circles*) compared to the fluorescence in a neighboring region (*open triangles*). (**h**) Integrated fluorescence recovery after photobleaching in regions of Rho^P23H^-EGFP that showed recovery following photobleaching (*c* and *d*). Averaged recovery profiles of photobleached areas in the two groups (*c* and *d*) were normalized in order to compare the half-time recovery of the mobile fraction of mutant opsin participating in recovery after photobleaching. Rho-EGFP (*red trace*) is included for comparison. (**i**) The photobleaching recovery profile of panel (e) from the ROI (*closed circles*) and a neighboring area (*open triangles*). There is no significant change in the fluorescence of the foci on the left of photobleaching box during the recovery period (*open triangles*). Scale bars, 5 µm.

We used fluorescent recovery after photobleaching (FRAP) in live retinal explants expressing Rho^P23H^-EGFP to characterize the mobility of the protein within the fluorescent foci. Both soluble EGFP and Rho-EGFP (Fig. 4) exhibited a single component fluorescence recovery profile after photobleaching expected for a mobile protein [18]. Since Rho-EGFP is restricted to movement within the disk, lateral recovery is the dominant factor in the FRAP signal. Thus, this diffusion occurs in the plane of the disk membrane and fluorescence recovery after photobleaching arises from fluorescent proteins diffusing laterally into the photobleached area. Under these experimental conditions, photobleaching of the targeted area (*white box* in Fig. 4 b–f) takes several seconds. The diffusion of fluorescent rhodopsin is rapid enough so that a decrease in the fluorescence density (∼30%) of the unbleached, laterally adjacent area is observed in the first scans after photobleaching (Fig. 4g, *neighboring area*). We note that the images are confocal scans from the photobleached optical plane; lateral diffusion of fluorescent rhodopsin also arises from fluorescent protein in front of and behind the bleached area.

We characterized Rho^P23H^-EGFP FRAP profiles of protein in three different regions: OS areas without dense protein-foci (Fig. 4c), IS (Fig. 4d) and OS (Fig. 4e,f) areas that contain fluorescent foci. The IS fluorescent foci are in the regions of the cell that contain both ER and Golgi (Fig. 1). To facilitate comparison between the rates of fluorescence recovery in these regions, we normalized the relative fluorescence changes in the photobleached areas (Fig. 4h). The average FRAP profile of Rho^P23H^-EGFP at the base of OS, where dense protein foci were not detectable, was similar to the profile found for Rho-EGFP (Fig. 4h). Moreover, the IS foci (Fig. 4d) had a fluorescence recovery in the photobleached area (Fig. 4h) and a decrement of fluorescence in the neighborhood of the photobleached area (Fig. 4d) similar to Rho-EGFP. These results show that, at least in photoreceptors, the Rho^P23H^-EGFP within IS foci is similar to wild type protein and more mobile than expected for protein found in aggresomes [3].

By contrast, areas with fluorescent foci in the OS showed no recovery from photobleaching (Fig. 4e, i), even though the foci were bleached in a way that left adjacent fluorescent regions intact. We found that highly fluorescent foci adjacent to a photobleached area did not exhibit any decrease in fluorescence, i.e. fluorescent protein in the foci was not able to move to a neighboring areas (Fig. 4e, i). These results indicate that the foci in the OS and IS have disparate physical properties and highlight that Rho^P23H^-EGFP has inherent mobility similar to Rho-EGFP when not found in fluorescent foci. This suggests either a change over time or in the cellular environment that causes the mutant protein to become immobile, e.g. aggregation or disk membrane breakdown.

**Figure 5:**
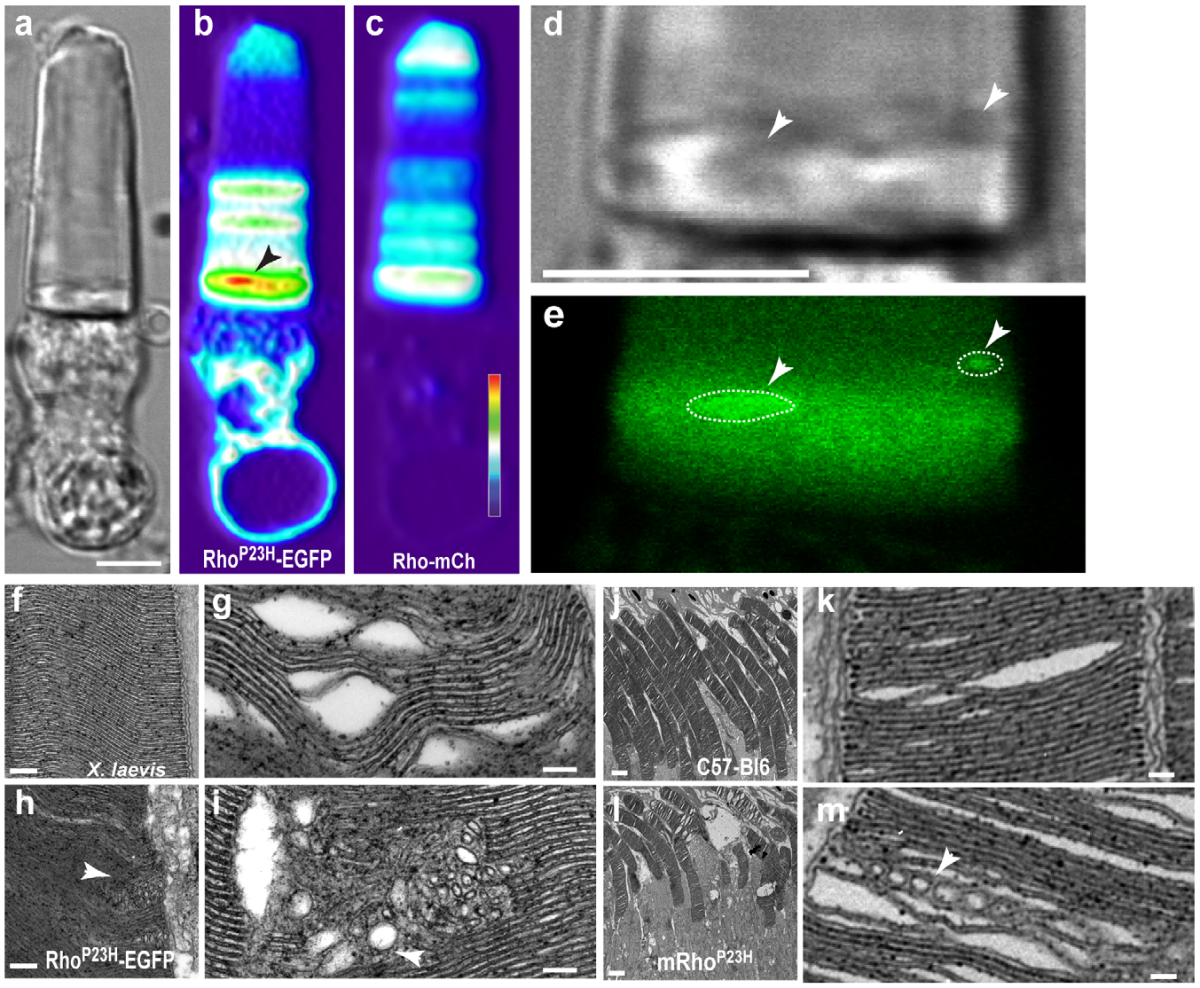
Defects in OS disk membranes in rods expressing Rho^P23H^-EGFP. (**a–e**) Images from a rod expressing fluorescent protein from two rhodopsin cassettes: Rho^P23H^-EGFP and Rho-mCherry (**b** and **c**, respectively). Both the heat map representation of Rho^P23H^-EGFP distribution (b, *arrow*) and reconstruction images of the cell fluorescence show Rho^P23H^-EGFP fluorescent foci (**e**, *arrow and dotted lines*) near the base of the OS that correspond to inhomogeneous OS disturbances of the DIC image (d, *arrows*). Scale bars (a–e), 5 µm. (**f–i**) Electron micrographs of rod OS from transgenic retina expressing either Rho-EGFP (f, g) or Rho^P23H^-EGFP (h, i). Membranes from Rho^P23H^-EGFP transgenic animals exhibited vesiculotubular structures (h, i, *arrowheads*) of similar size to the fluorescent foci found in CSLM imaging. These structures were not observed in micrographs from Rho-EGFP transgenic animals (f). Mechanical disruption of retina prior to fixation (g) showed OS breaks and disk membrane separations but no vesiculotubular structures. Micrographs from four animals were examined for each group. (**j–m**) Electron micrographs of retina from wild-type (j) or Rho^P23H^ (GHL) transgenic mice (l). Membranes from Rho^P23H^ transgenic animals exhibited vesiculotubular structures (m, *arrowheads*). Vesiculotubular structures were not observed in micrographs from wild type animals (j, k). Scale bars, 100 nm (g, i, *k, m*), 200 nm (*f, h*) and 2 µm (j, l).

Cells expressing Rho^P23H^-EGFP with fluorescent foci often exhibited disturbances of the OS in differential interference contrast micrographs (Fig. 5a, d). The disturbances coincided with foci (Fig. 5b, e) and were not observed in OS containing Rho-EGFP or Rho^P23H^-EGFP without foci. OS morphology of transgenic animals that express Rho-EGFP was indistinguishable from non-transgenic animals, exhibiting many closely stacked disks in electron micrographs (Fig. 5f). However, OS containing mutant opsin had vesiculotubular structures (VT) between 50–400 nm in diameter in a single section (Fig. 5h, i). The continuity of underlying disk membranes was totally disrupted around the VTs (Fig. 5i). These were observed in 11 of the 70 cells examined from 4 animals. During preparation for electron microscopy, tissue damage sometimes occurred, causing breaks or distortions in the outer segment (Fig. 5g). These defects were distinct and easily distinguished from the VTs. We also examined a mouse line containing a Rho^P23H^ transgene [8] and found similar VT structures (Fig. 5l, m). These results provide strong evidence for a common process underlying OS disruption in vertebrates. Furthermore, they suggest that Rho^P23H^ functions at low concentrations compared to wild type protein, catalyzing local changes in disk membranes, possibly through homo-oligomerization. Future work should be directed toward establishing a molecular mechanism for this process.

**Figure 6:**
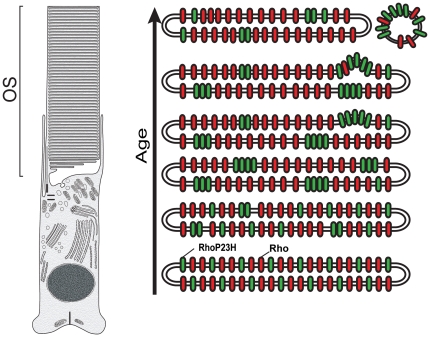
Schematic model to explain OS defect formation in disks that express mutant opsin. Wild type rhodopsin (*red*) distributes randomly throughout the disk membrane (except incisures which are not shown). In rods expressing Rho^P23H^ (*green*), there is an initial random distribution of mutant with wild type protein in the disk, although the concentration of mutant protein is much lower than wild type protein. According to this model, over time, the Rho^P23H^ mutant begins to self-associate and form aggregates in the membrane, excluding wild type protein. The resulting mutant protein concentrates in a localized area that causes deformation or defects in the membrane structure, leading to vesiculation and disk breakdown. This could lead to structural instability in the OS, initiating a breakdown and potentially rod death.

Previous work has reported disruptions of degenerating OS in mice expressing a Rho^P23H^ transgene [7]–[9]. Abnormal OS membranes are also formed in *Xenopus* retina treated with tunicamycin [19] and in rats deprived of vitamin A [20]. However, the underlying causes have not been established. This report established that low level expression of mutant opsin leads to a destructive effect on disk membranes via subcellular microstructures. We hypothesize that the mutant protein undergoes self-aggregation with age (Fig. 6). Apparently, either the Rho^P23H^-EGFP is not present in the IS long enough to form immobile aggregates or there are factors not present in the OS that prevent their formation. It is possible that misfolded opsin, *i.e.* protein that cannot bind 11-*cis* retinal, is responsible for fluorescent foci formation. However, since Rho^P23H^ retains the ability to bind 11-*cis* retinal [21], additional experiments using other mutants with more severe defects (e.g. Rho^C187R^-EGFP) will be necessary to clarify this point. Our results highlight that even with a robust protein quality control response that leads to a decrease in mutant protein expression, small amounts of mutant protein that escape the ER and are transported to the OS can have a deleterious effect on the cell. As such, this report proposes a novel mechanism for the toxic effect of opsin mutants. The damaged disk membranes are potentially more susceptible to mechanical injury or instability, resulting in their collapse.

Autosomal dominant RP is a slow disease [22], in which rods may up-regulate the UPR [4] and degrade mutant opsin (including aggregates in the IS). However, over time, some mutant opsin may escape to the OS and undergo a time-dependent aggregation leading to VT formation. Daily disk shedding will remove defective membrane and reduce potential toxicity, acting as another quality control step. Eventually, VTs may persist and cause mechanical breakdown. This process of membrane destabilization may also be relevant to Alzheimer's disease and other common neurodegenerative diseases [16].

## Materials and Methods

### Transgenic animals

Animals expressing rhodopsin transgenes in rods were produced by restriction enzyme mediated integration into *Xenopus* sperm nuclei as previously described with modifications [23]–[26]. Plasmids containing transgenes were linearized with XhoI or NheI (New England Biolabs) and purified prior to injection. GFP expression in live tadpoles was estimated using a fluorescence dissecting microscope (Leica MZ FL III) and divided into three intensity groups (bright, medium and faint) according to an arbitrary scale based upon photographic exposure times, using soluble GFP for comparison. Animals were maintained on a 12 h-12 h light-dark cycle at 18–20°C. All animal handling and experiments were in agreement with the animal care and use guidelines of the Association for Research in Vision and Ophthalmology (ARVO). This study was done under the approval of the SUNY Upstate Medical University Committee on the Human Use of Animals (CHUA no. 209).

### Transgenic DNA constructs

The Rho-EGFP cDNA encoded amino acids 1–354 of *Xenopus* rhodopsin [27] fused in frame with EGFP followed by the 1D4 epitope (ETSQVAPA) [28] at the carboxyl terminus [29]. Mutations were introduced into the Rho-EGFP cDNA using QuikChange II XL (Stratagene). The promoter used in all experiments was a fragment containing nucleotides −503 to +41 from the *Xenopus* opsin (XOP) gene [31]. Dual transgene expression constructs consisted of two XOP(−503/+41)-Rho-EGFP/mCherry (or Rho^P23H^-EGFP/mCherry) cassettes in tandem. All protein coding regions were sequenced on both strands and then linearized with XhoI or NheI prior to transgenesis.

### Immunohistochemistry and electron microscopy

For immunohistochemistry, eyes were fixed in 4% paraformaldehyde in PBS at 4°C and frozen in OCT (TissueTek Inc.). Frozen blocks were sectioned at 12–20 µm slices with a Microm 560 cryostat (Richard Allen Scientific) and immunostained with primary antibody overnight at 4°C, washed and then incubated with appropriate conjugated secondary antibody. Primary antibodies used were mouse anti-rhodopsin 4D2 (1∶3000) and anti-mouse secondary antibody (Jackson ImmunoResearch) conjugated to Cy3 (1∶500). Slides were examined with the *i*-80 series (Nikon) or LSM-510 confocal microscopes (Zeiss). For preparation of EM sections, eyes were fixed in 2.5% glutaraldehyde and 1% OsO4 and embedded in epoxy resin.

### Total RNA extraction and qPCR

Six retina without retinal pigmented epithelium were isolated and used for total RNA extraction. cDNA was generated using QuantiTect™ Reverse Transcription Kit (Qiagen) and real time PCR was performed using SYBR Green MasterMix I and the LightCycler® 480 Real-Time PCR System (Roche Applied Science). Data were analyzed by the LightCycler® software and plotted using Sigmaplot (Systat).

### Live cell imaging

The recording chamber was made in the center of a 5 cm plastic Petri dish as described previously [30]. The bottom of the chamber was comprised of a number 1 coverslip attached to the bottom of the chamber permitting the access of the microscope lenses. Retinal explants were minced into small pieces and placed into the chamber with Ringer's solution (in mM: NaCl 111, KCl 2, CaCl_2_ 1, MgCl_2_ 1, MgSO_4_ 0.5, NaH_2_PO_4_ 0.5, HEPES 3, glucose 10, EDTA 0.01). The chamber was then covered by a number 1 coverslip and placed onto the microscope stage for imaging. For quantification of foci, seven tadpoles from two transgenic lines expressing mutant opsin (RhoP23H-EGFP) were examined by live imaging. Data was collected from 17 different retinal explants (∼70% exhibited foci). 41 out of 254 cells explants demonstrated fluorescent foci.

### Quantification of proteins by confocal microscopy

Images for quantification of transgenic proteins expressed in rod photoreceptors were acquired using the LSM-510 software driving a confocal LSM-510 imaging system (Zeiss). Retinal explants were immersed in frog Ringer's solution and scanned by a laser line of 488 or 543 nm. The scanning objective for both channels was a Plan-Neofluar 63×/1.4 oil lens (Zeiss). To minimize the bleeding through channels, the emitted light from 488 nm excitation was filtered by a 500–535 nm filter, and the emitted light from the 543 nm excitation was filtered by a 655–710 band pass filter. The resolution of all scanned images was set to 0.04×0.04 µm in the *xy* plane. At least 5 *z* scans from the central area of the rod photoreceptor with a 0.5 µm interval were obtained. The LSM-510 software was set to correct for the *z* plane while scanning dual channels. The pixel time, pinhole diameter, amplifier offset and amplifier gain for all scans were set to 3 µs, 1.4 airy units, 0.1 and 1, respectively. Recombinant EGFP and mCherry at different concentrations were scanned at the same settings used for retinal explant imaging (Fig. S3).

### Image analysis for protein quantification

Fluorescent intensities of images were measured using AxioVision™ software version 4.7 (Zeiss), corrected for the background intensity, and averaged values of at least three *z* sections were used for protein measurements. The measured fluorescent intensities were converted to protein concentration using values obtained from scanned fluorescent solutions containing known concentrations of recombinant fluorescent proteins (Fig. S3).

### FRAP analysis by confocal microscopy

For acquisition of images in FRAP experiments the LSM software version 2.8 was used to drive a LSM-510 confocal microscope. Retinal explants were prepared as previously described and imaged using a Plan-Neofluar 63×/1.4 oil lens (Zeiss). The laser line for scanning with 488 nm light powered by an Argon laser was set at 21 milliwatt. The laser power for image acquisition before and after photobleaching was set to 0.5–4% of the maximum laser power and to 100% during photobleaching. The recording chamber was initially searched for retinal explants having rods which were parallel with the covering coverslip. A single rod photoreceptor was centered in the imaging window and 8 bit images in gray scale 255 were scanned usually from a square, 13.2×13.2 µm in *xy* plane, or 256×256 pixels. The photobleaching was performed in the central part of the OS or within dense fluorescent foci found in the IS in a rectangular region 1.8 µm in diameter and usually for 40 ms/µm^2^ per iteration. The laser scanning performed before photobleaching, during photobleaching and after, was in the same *z* axis. The images in the recovery phase were taken right after photobleaching and then every 3–5 seconds in time series of at least 25 recovery scans. FRAP data were analyzed using AxioVision™ software version 4.7 (Zeiss) and SigmaPlot™ (Systat Software Inc.). Briefly, the images were corrected for background fluorescence and the averaged intensity of the selected area was measured (AxioVision™) and plotted. The averaged intensity of the recovering area was normalized to the non-bleached neighboring area in each scanned image. The time for recovery to 50% of maximum fluorescence (t_1/2_) was measured by fitting a one parameter exponential function using SigmaPlot. The error bars show the SEM.

## Supporting Information

Figure S1 **Expression of Rho^P23H^-EGFP in transgenic** ***Xenopus*** **.** (**a–d**) Immunohistochemistry of fixed frozen sections of transgenic tadpoles (approximately stage 45) with nuclei stained with DAPI (*blue*) to identify retinal layers and anti-rhodopsin-Cy3 secondary antibody (*red*) to detect both endogenous and transgenic opsin. A retina from an animal with high expression of Rho-EGFP (a) has strong green fluorescence throughout the photoreceptor layer (*ONL*). There is a corresponding strong staining with anti-rhodopsin antibody observable in a merged image (a, *right*), where most OS appear to have both EGFP and Cy3 fluorescence (*yellow*). A higher magnification (b) of the boxed region of (a) shows prominent fluorescence in the OS and a minor amount in the IS-synaptic regions (*dotted arrow*). (c) A retina from an animal with high expression of Rho^P23H^EGFP has weak, punctate green fluorescence throughout the photoreceptor layer (*ONL*) and also in the RPE (c, *middle*). OS are degenerated, but the cell number based upon DAPI staining is comparable to wild type animals. Scale bar, 50 µm. (d) A higher magnification of the boxed area (c) is shown (*left*). There is only one OS in this section (*arrow*) which appears shortened and disorganized, and a few surviving cones with OS (*yellow arrowheads*). There are three regions containing EGFP fluorescence (*middle*). The sole OS exhibits EGFP fluorescence. In addition, there is an abnormal localization of EGFP fluorescence in the ONL/IS region (IS) which cross-reacts with anti-rhodopsin antibodies (d, *right*). Finally, there is strong fluorescence from both EGFP and anti-rhodopsin staining in the RPE (*asterisk*). There is no fluorescence in the RPE in retina expressing Rho-EGFP (b, *right*). Scale bar, 10 µm. Blue, DAPI, Lens, L, retinal pigment epithelium, RPE.
(TIF)

Figure S2 **Quantification of transgene expression from dual rhodopsin cassettes in** ***Xenopus*** **retina.** Quantification of Rho-EGFP and Rho-mCherry transcripts by real time PCR in eyes (n = 6) from transgenic tadpoles harboring one plasmid with two cassettes, each with a *Xenopus* opsin promoter driving the expression of Rho-EGFP (*green circle*) and Rho-mCherry (*red circle*). The difference between Rho-EGFP and Rho-mCherry was 0.2±0.15 fold (mean ± SD), thus demonstrating that the dual cassette construct produces equimolar concentrations of transcripts. The transgene transcripts were less than 1/1000 of the endogenous rhodopsin level (*triangle*).
(TIF)

Figure S3 **Calibration of the fluorescence intensity by recombinant EGFP and mCherry.** Calibration of the fluorescence intensity from EGFP and mCherry in the CSLM chamber using different concentrations of recombinant EGFP and mCherry. The laser power and detector sensitivity were identical for all measurements (error bar, SEM).
(TIF)

Figure S4 **Rho-EGFP concentration is significantly reduced in OS disk incisures compared to OS membranes.** (**a**) 3D confocal and corresponding DIC image of a live photoreceptor expressing Rho-EGFP. Regions with reduced fluorescence intensity (*arrows*) extend along the axial length of the OS in disk incisures, spanning across the diurnal transverse variations. (**b**) Four sequential images of optical *z*-sections obtained parallel to the rod axis show that the Rho-EGFP exclusion is more prominent in the periphery and maintains continuity with incisures from the base to the tip of OS. The exclusion zones are regularly spaced around the circumference of the OS, similar to the incisure pattern. (**c**) Representative cross-sectional view of an OS from a live rod expressing Rho-EGFP. The *z*-section for the CSLM was parallel to the rod axis and shows regions of reduced fluorescence intensity (*arrows*) that extend from the OS periphery toward the center. (**d**) A cross-sectional electron micrograph of a rod shows disk incisures which resemble the regions of Rho-EGFP exclusion (c). Scale bar, 5 µm (a, b) and 1 µm (c,d).
(TIF)

Figure S5 **Fluorescent foci formed by Rho^P23H^-mCherry transgene in transgenic rods.** (**a**) A CSLM image of a live retinal explant from an animal harboring both Rho-EGFP and Rho^P23H^-mCherry transgenes. Two cells have expressed Rho-EGFP and one has also expressed Rho^P23H^-mCherry. A DIC image of photoreceptors overlaid on both green and red channels is shown (*right*). Fluorescent foci (*arrows*) in the mCherry images are apparent in the longitudinal scan. Note that the foci have formed during a period in which the Rho-EGFP expression is less than the Rho-mCherry. (**b**) A single optical section from an end-on CSLM scan from a cell expressing both Rho-EGFP and Rho^P23H^-mCherry transgenes. Foci (*arrow*) are present in the red but not green channel. A DIC image of photoreceptors overlaid on both green and red channels is shown (*right*). Scale bar, 5 µm.
(TIF)
