# Supplementary figures and images for: Rhodopsin Mutant P23H Destabilizes Rod Photoreceptor Disk Membranes
Source: PLoS One. 2012 Jan 19;7(1):e30101. doi: 10.1371/journal.pone.0030101 (PMC3261860; doi:10.1371/journal.pone.0030101)

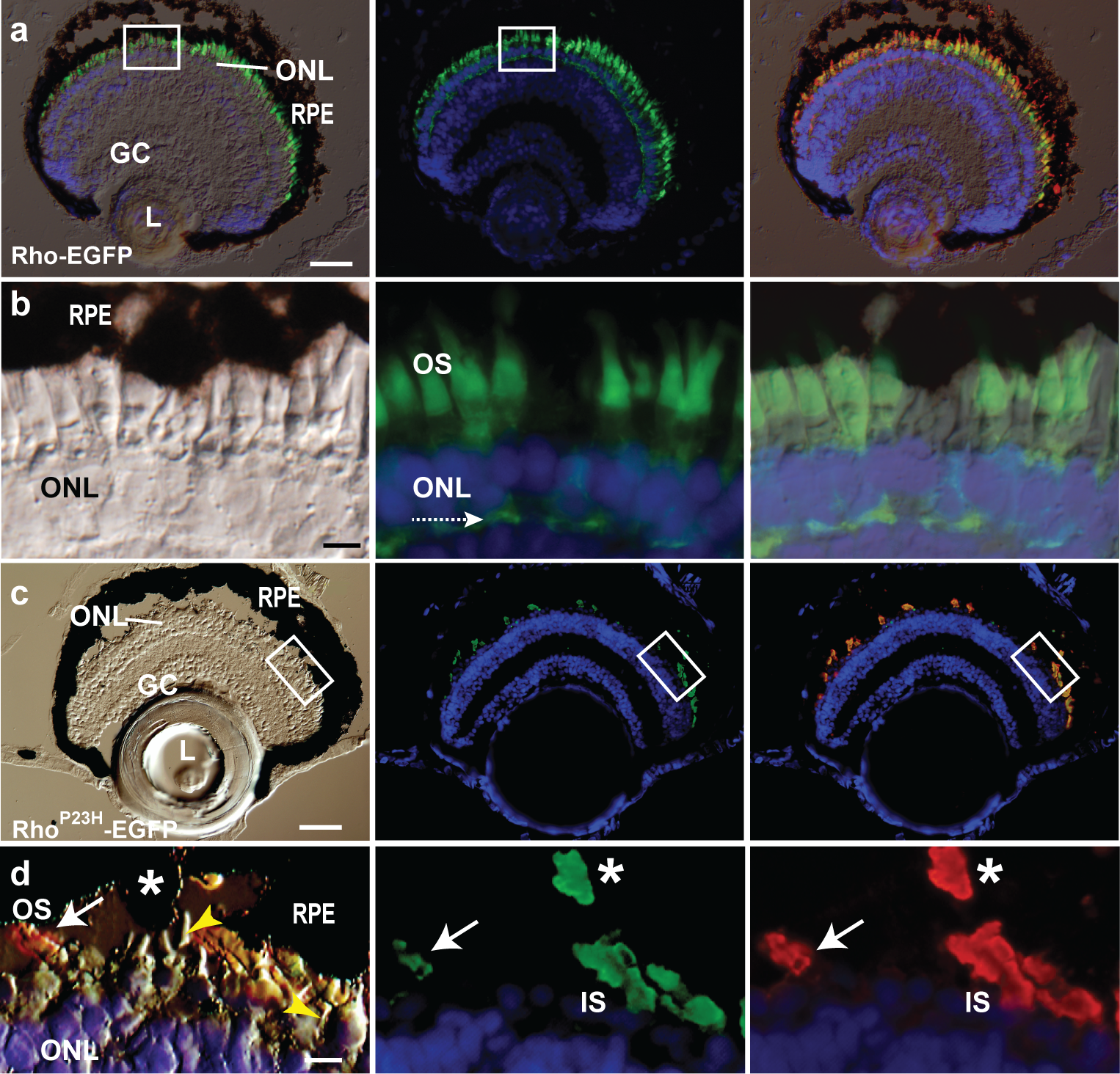

Supplement: Figure S1 — Expression of RhoP23H-EGFP in transgenic Xenopus . (a–d) Immunohistochemistry of fixed frozen sections of transgenic tadpoles (approximately stage 45) with nuclei stained with DAPI (blue) to identify retinal layers and anti-rhodopsin-Cy3 secondary antibody (red) to detect both endogenous and transgenic opsin. A retina from an animal with high expression of Rho-EGFP (a) has strong green fluorescence throughout the photoreceptor layer (ONL). There is a corresponding strong staining with anti-rhodopsin antibody observable in a merged image (a, right), where most OS appear to have both EGFP and Cy3 fluorescence (yellow). A higher magnification (b) of the boxed region of (a) shows prominent fluorescence in the OS and a minor amount in the IS-synaptic regions (dotted arrow). (c) A retina from an animal with high expression of RhoP23HEGFP has weak, punctate green fluorescence throughout the photoreceptor layer (ONL) and also in the RPE (c, middle). OS are degenerated, but the cell number based upon DAPI staining is comparable to wild type animals. Scale bar, 50 µm. (d) A higher magnification of the boxed area (c) is shown (left). There is only one OS in this section (arrow) which appears shortened and disorganized, and a few surviving cones with OS (yellow arrowheads). There are three regions containing EGFP fluorescence (middle). The sole OS exhibits EGFP fluorescence. In addition, there is an abnormal localization of EGFP fluorescence in the ONL/IS region (IS) which cross-reacts with anti-rhodopsin antibodies (d, right). Finally, there is strong fluorescence from both EGFP and anti-rhodopsin staining in the RPE (asterisk). There is no fluorescence in the RPE in retina expressing Rho-EGFP (b, right). Scale bar, 10 µm. Blue, DAPI, Lens, L, retinal pigment epithelium, RPE. (TIF) [file pone.0030101.s001.tif]

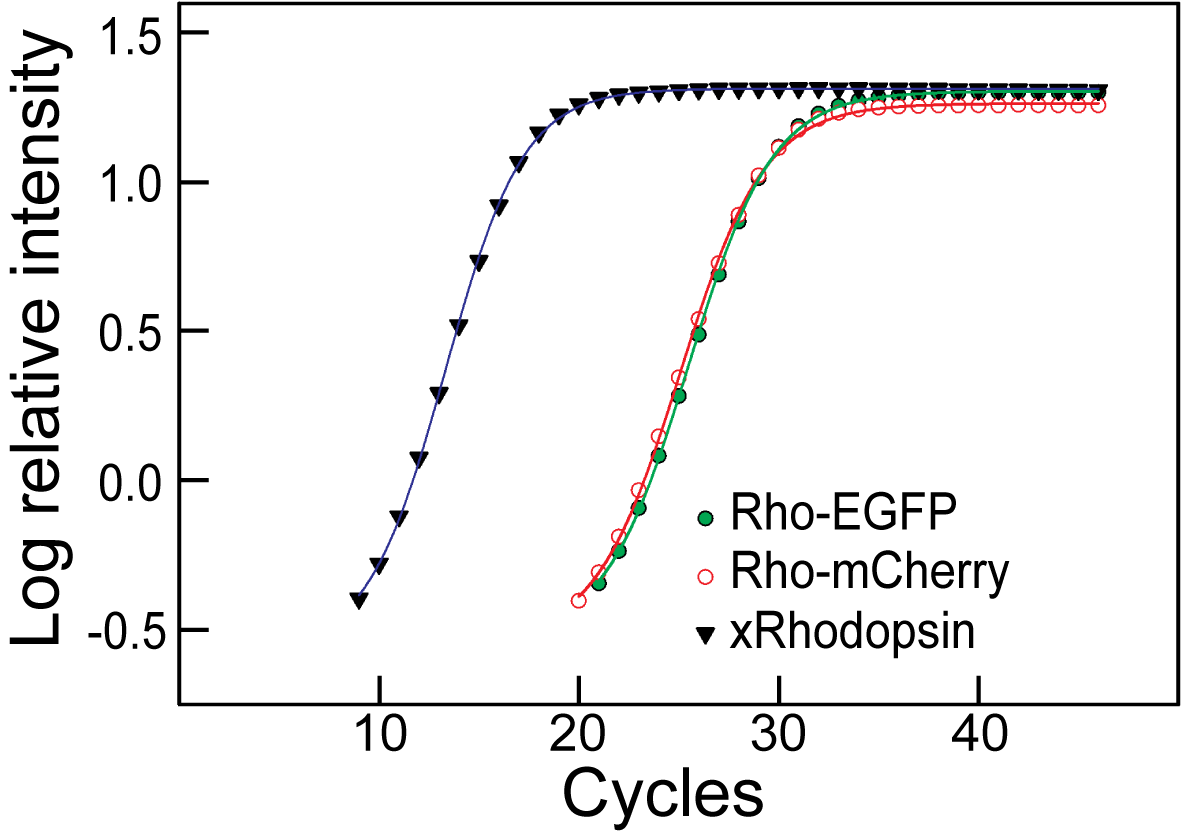

Supplement: Figure S2 — Quantification of transgene expression from dual rhodopsin cassettes in Xenopus retina. Quantification of Rho-EGFP and Rho-mCherry transcripts by real time PCR in eyes (n = 6) from transgenic tadpoles harboring one plasmid with two cassettes, each with a Xenopus opsin promoter driving the expression of Rho-EGFP (green circle) and Rho-mCherry (red circle). The difference between Rho-EGFP and Rho-mCherry was 0.2±0.15 fold (mean ± SD), thus demonstrating that the dual cassette construct produces equimolar concentrations of transcripts. The transgene transcripts were less than 1/1000 of the endogenous rhodopsin level (triangle). (TIF) [file pone.0030101.s002.tif]

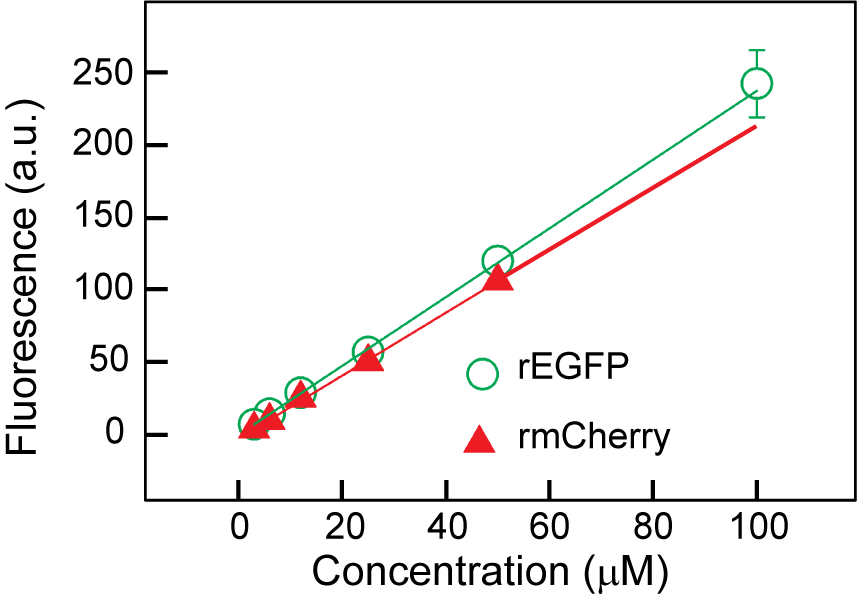

Supplement: Figure S3 — Calibration of the fluorescence intensity by recombinant EGFP and mCherry. Calibration of the fluorescence intensity from EGFP and mCherry in the CSLM chamber using different concentrations of recombinant EGFP and mCherry. The laser power and detector sensitivity were identical for all measurements (error bar, SEM). (TIF) [file pone.0030101.s003.tif]

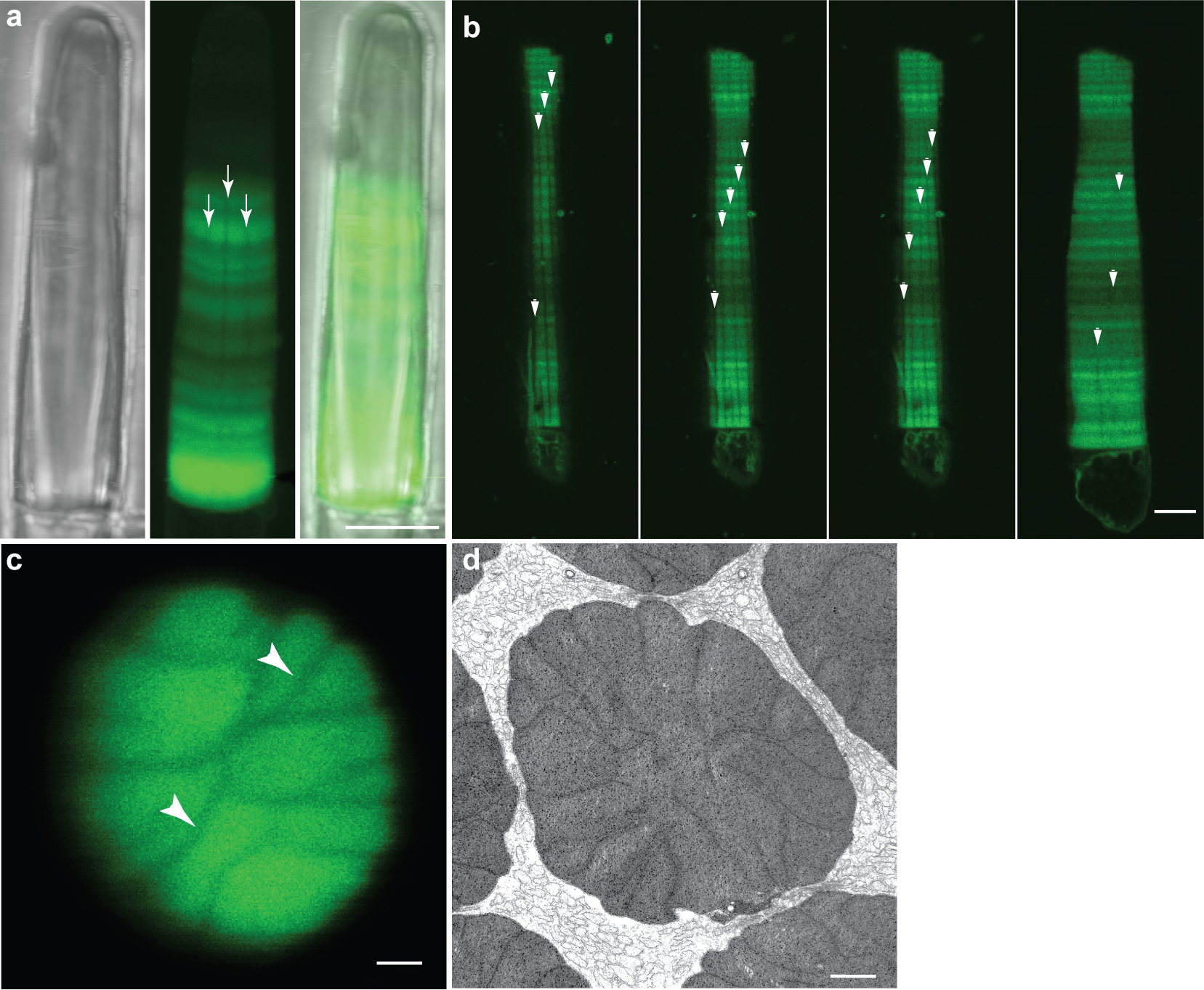

Supplement: Figure S4 — Rho-EGFP concentration is significantly reduced in OS disk incisures compared to OS membranes. (a) 3D confocal and corresponding DIC image of a live photoreceptor expressing Rho-EGFP. Regions with reduced fluorescence intensity (arrows) extend along the axial length of the OS in disk incisures, spanning across the diurnal transverse variations. (b) Four sequential images of optical z-sections obtained parallel to the rod axis show that the Rho-EGFP exclusion is more prominent in the periphery and maintains continuity with incisures from the base to the tip of OS. The exclusion zones are regularly spaced around the circumference of the OS, similar to the incisure pattern. (c) Representative cross-sectional view of an OS from a live rod expressing Rho-EGFP. The z-section for the CSLM was parallel to the rod axis and shows regions of reduced fluorescence intensity (arrows) that extend from the OS periphery toward the center. (d) A cross-sectional electron micrograph of a rod shows disk incisures which resemble the regions of Rho-EGFP exclusion (c). Scale bar, 5 µm (a, b) and 1 µm (c,d). (TIF) [file pone.0030101.s004.tif]

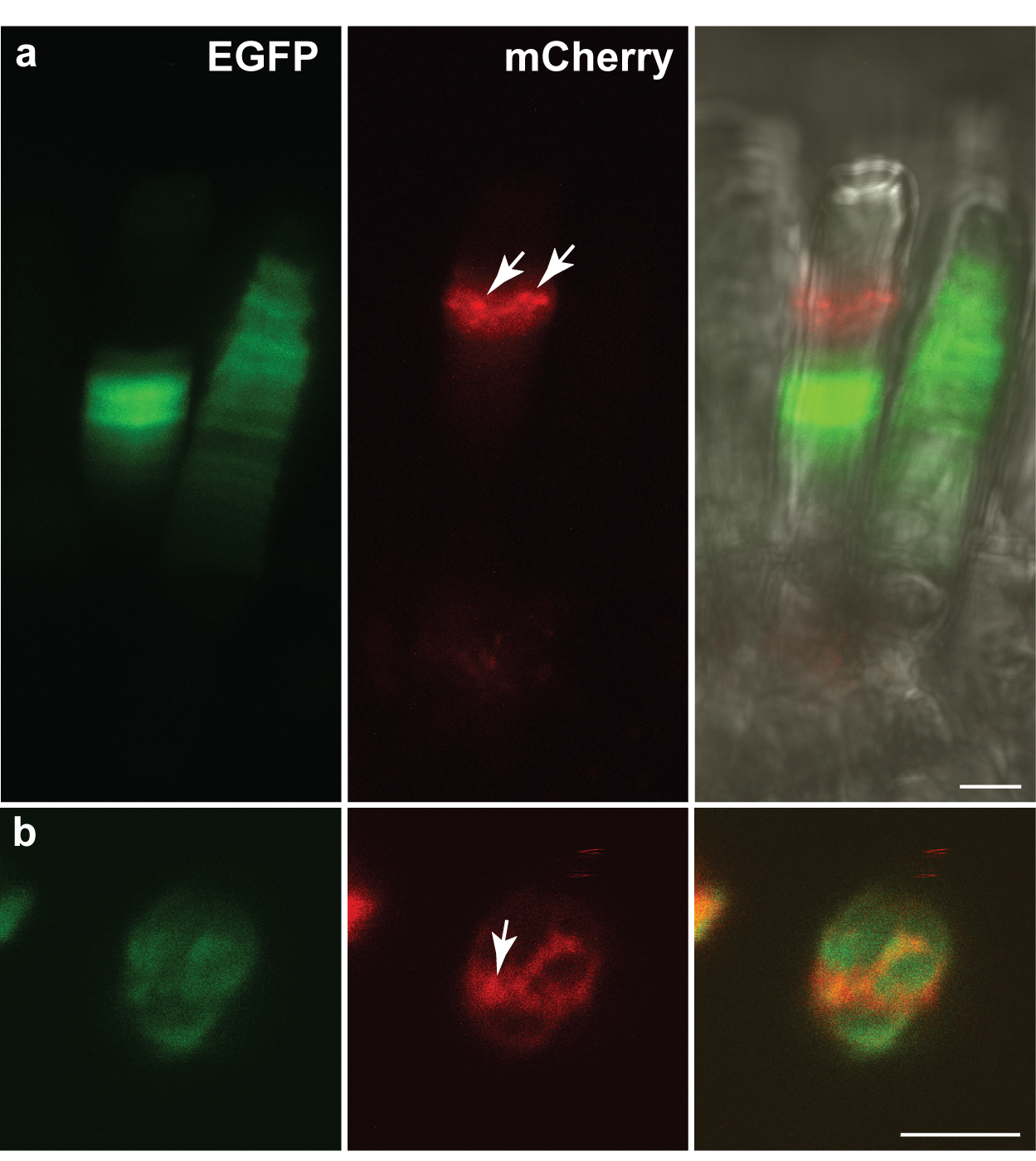

Supplement: Figure S5 — Fluorescent foci formed by RhoP23H-mCherry transgene in transgenic rods. (a) A CSLM image of a live retinal explant from an animal harboring both Rho-EGFP and RhoP23H-mCherry transgenes. Two cells have expressed Rho-EGFP and one has also expressed RhoP23H-mCherry. A DIC image of photoreceptors overlaid on both green and red channels is shown (right). Fluorescent foci (arrows) in the mCherry images are apparent in the longitudinal scan. Note that the foci have formed during a period in which the Rho-EGFP expression is less than the Rho-mCherry. (b) A single optical section from an end-on CSLM scan from a cell expressing both Rho-EGFP and RhoP23H-mCherry transgenes. Foci (arrow) are present in the red but not green channel. A DIC image of photoreceptors overlaid on both green and red channels is shown (right). Scale bar, 5 µm. (TIF) [file pone.0030101.s005.tif]
